# Effect of drug interactions with apixaban on clinical outcomes in cancer patients with venous thromboembolism

**DOI:** 10.3389/fonc.2025.1520725

**Published:** 2025-01-27

**Authors:** Marte Svalastoga, Trine-Lise Larsen, Jorunn Brekke, Tone Enden, Hege Frøen, Herish Garresori, Eva Marie Jacobsen, Alina Carmen Porojnicu, Anne Hansen Ree, Dag Torfoss, Elin Osvik Velle, Hilde Skuterud Wik, Waleed Ghanima, Per Morten Sandset, Anders Erik Astrup Dahm

**Affiliations:** ^1^ Institute of Clinical Medicine, University of Oslo, Oslo, Norway; ^2^ Department of Haematology, Akershus University Hospital, Lørenskog, Norway; ^3^ Department of Oncology, Haukeland University Hospital, Bergen, Norway; ^4^ Department of Radiology, Oslo University Hospital, Oslo, Norway; ^5^ Department of Medicine, Baerum Hospital, Vestre Viken Hospital Trust, Drammen, Norway; ^6^ Department of Oncology, Stavanger University Hospital, Stavanger, Norway; ^7^ Department of Haematology, Oslo University Hospital, Oslo, Norway; ^8^ Department of Oncology, Drammen Hospital, Vestre Viken Hospital Trust, Drammen, Norway; ^9^ Department of Oncology, Akershus University Hospital, Lørenskog, Norway; ^10^ Department of Oncology, Oslo University Hospital, Oslo, Norway; ^11^ Department of Medicine, Volda Hospital, Møre and Romsdal Hospital Trust Volda, Volda, Norway; ^12^ Clinic of Internal Medicine, Østfold Hospital, Grålum, Norway

**Keywords:** venous thromboembolism, apixaban, cancer, drug-drug interaction, bleeding

## Abstract

**Introduction:**

It is unclear how drug-interaction with apixaban influences recurrent venous thromboembolism (VTE) and bleedings in cancer patients.

**Methods:**

A *post-hoc* analysis of a single-arm interventional clinical trial on apixaban treatment of cancer patients with VTE to investigate whether the occurrence of any of the endpoints could be associated with the concurrent use of an interacting drug. Drugs taken by the patients during the trial period were categorized as either increasing bleeding risk, increasing thrombosis risk, both or neither.

**Results:**

298 patients were divided into groups based on whether they used no interacting drugs (controls, n=74), drugs increasing bleeding risk (n=55), drugs increasing thrombosis risk (n=8), or both (n=161). Odds ratios (OR) were calculated for recurrent VTE, clinically relevant non-major bleeding (CRNMB), and major bleeding during the 36-month follow-up period. Each patient took a median of 13 different drugs over the study period. 67% of the patients used drugs expected to both increase bleeding and thrombosis. The use of fluconazole appeared associated with CRNMB (OR 3.6, 95% confidence interval (CI) 0.99-13), but not with major bleeding (OR 0.56, 95% CI 0.06 - 4.8). Non-steroid anti-inflammatory drugs were not associated with CRNMB (OR 1.0, 95% CI 0.25-4.1) or major bleedings (OR 0.72, 95% CI 0.14 - 3.6). Use of antiplatelet therapy was not associated with CRNMB (OR 0.75, 95% CI, 0.22 - 2.58) or major bleeding (OR 0.2, 95% CI, 0.02-1.6). There were no major bleedings in 23 patients using aprepitant nor in the 10 patients taking macrolides. We found no association between drugs and recurrent VTE, except that there were no recurrent VTE in 19 patients using bevacizumab.

**Conclusions:**

Despite the high number of drugs taken that could potentially interact with apixaban, none were found to clearly influence clinical outcomes, except that fluconazole may increase the risk of CRNMB.

## 1 Introduction

Patients with cancer are known to be at higher risk of venous thromboembolism (VTE) than the general population (1, 2). In recent years, several studies have shown that direct-acting oral anticoagulants (DOACs) edoxaban, rivaroxaban, and apixaban are as effective as low-molecular-weight heparin (LMWH) in prevention of recurrent thrombosis. DOACs were generally found to be non-inferior to LMWH (3–6), and are currently an alternative to LMWH for cancer patients (7). Apixaban is a substrate of cytochrome P450 3A4 (CYP3A4) (8) and permeability glycoprotein 1 (P-gp) (9), which makes it susceptible to pharmacokinetic interactions with inducers or inhibitors of these pathways. Such interaction can reduce or increase the plasma concentration of apixaban, respectively. Accordingly, DOACs are not recommended for cancer patients taking anticancer therapies that significantly affect these pathways (10). Less is, however, known about how changes in the plasma concentration translate into clinical outcomes, and how real-world combinations of both inducers and inhibitors of CYP3A4 and/or P-gp affect the efficacy of apixaban.

In addition to drugs with pharmacokinetic interactions, many drugs commonly taken by cancer patients can increase the risk of bleeding or thrombosis in apixaban users through pharmacodynamic interactions, by directly influencing the hemostasis. Examples are the increased risk of bleeding seen with non-steroid anti-inflammatory drugs (NSAIDs) or platelet inhibitors, and the increased thrombosis risk associated with antihormonal treatments like tamoxifen (11, 12).

A systematic review of drug-drug interactions with DOACs found that interactions between amiodarone and dabigatran were the most frequently associated with bleeding events (13). Amiodarone, fluconazole, rifampicin, and phenytoin were found to increase the risk of major bleeding when taken in combination with one of the DOACs dabigatran, rivaroxaban, or apixaban (14). Hanigan et al. reported a trend towards increased bleeding risk in patients taking rivaroxaban or apixaban combined P-gp and CYP3A4 inhibitors like amiodarone, dronedarone, diltiazem, and verapamil (15).

In a *post-hoc* analysis of the Caravaggio study, which compared dalteparin with apixaban in cancer patients with VTE, no association was found between anti-cancer treatment and the risk of recurrent VTE or major bleeding (16). Likewise, drug-drug interactions did now influence recurrent VTE or major bleeding in a cohort of patients treated for cancer (17).

The aim of the current report is to describe how drugs expected to interact with apixaban influenced recurrent VTE and bleedings in cancer patients participating in a multicenter single-arm interventional study of apixaban (CAP study) (18).

## 2 Materials and methods

A *post-hoc* analysis was be performed on data collected from the CAP study, which was originally designed to investigate the safety and efficacy of apixaban for treating VTE in cancer patients. The study was carried out from April 2016 until May 2018, and included 298 patients across nine Norwegian hospitals. The inclusion and exclusion criteria for the study have previously been reported (18). In short, the patients were 18 years or older and either had a diagnosis of cancer or were treated for cancers other than basal cell or squamous cell carcinoma of the skin, had an objectively verified VTE, and did not use strong inhibitors or inducers of CYP3A4 or P-gp. Interacting drugs that were not allowed in the study according to the protocol were voriconazole, posaconazole, itraconazole, idelalisib, several drugs against HIV and hepatitis C, phenytoin, carbamazepine, phenobarbital, primidone, enzalutamide, dabrafenib, and rifampicin. Drugs that were considered moderate interactors or with an unknown degree of interaction were allowed. The patients received apixaban 10 mg twice daily the first week, then 5 mg twice daily for 6 months. If there were indication of continued anticoagulation after 6 months, they received apixaban 2.5 mg twice daily. Clinical endpoints and a complete list of medications taken were registered at inclusion, after 3, 6, and 12 months, and then every 12^th^ month for a total of 36 months. Ethics approval was obtained from the Norwegian Regional Committees for Medical and Health Research Ethics and the local data protection officer at each participating hospital. The study was carried out in accordance with the principles of the Declaration of Helsinki and Good Clinical Practice.

### 2.1 Categories of interacting drugs

Interactions of both *pharmacokinetic* (mainly through CYP3A4 or P-gp interference) and *pharmacodynamic* nature were considered, i.e. drugs with a known effect on hemostasis, either as effects or side effects. For the pharmacokinetic interactions, categorization of drugs as either strong, moderate, or weak inducers or inhibitors of CYP enzymes was based on FDA’s 2020 Guidance for Industry on clinical drug interaction studies (19), summarized in **Table 1**. The guidance used measured differences in area under concentration-time curve (AUC) when an index substrate was combined with an interactor to describe the magnitude of effect.

**Table 1:** Definitions of weak to strong inhibitors and inducers of CYP enzymes, from FDA’s 2020 Guidance for Industry on clinical drug interaction studies (19).

Category	Effect on AUC of index substrate
Strong inhibitor	Increased ≥ 5-fold
Moderate inhibitor	Increased ≥2- to <4-fold
Weak inhibitor	Increased ≥1.25- to <2-fold
Strong inducer	Decreased by ≥ 80%
Moderate inducer	Decreased by ≥50% to <80%
Weak inducer	Decreased by ≥20% to <50%

While drugs with strong pharmacokinetic interactions with apixaban were exclusion criteria for the study, the study population could use mild/moderate interactors, as well as some groups of medications that increase the risk of bleeding and/or thrombosis. Lists of potentially interacting drugs were sourced from previous reports on the topic (15, 20), and the appendix of the initial report from the CAP study (18). All patients that had taken at least one dose of an interacting drug during the study were considered users of that drug.

Potentially interacting drugs were categorized as: i) drugs where a higher risk of bleeding was assumed, either because the drugs inhibit the metabolism of apixaban, thus potentially increasing its potency, or because of other features, e.g., platelet inhibition; ii) drugs assumed to have a higher risk of thrombosis, either because of induction of the metabolic paths of apixaban, or because of other features, e.g., cisplatin; iii) drugs with a combination of interactions or effects from both previous groups.

Patients were correspondingly categorized as taking drugs from the first group “expected only increased bleeding risk”, the second group “expected only increased thrombosis risk”, or from the third group “expected both increased bleeding risk and thrombosis risk”. Patients taking drugs from both the first and second group were placed in the third group. Patients not exposed to drugs in either of the three interacting drug groups served as control group. For each group, odds ratio (OR) for each of the outcomes were calculated and compared with the control group.

### 2.2 Outcomes

Three main clinical outcomes were defined in the CAP study for the efficacy and safety assessments of apixaban: recurrent VTE, major bleeding, and clinically relevant non-major bleeding (CRNMB).

Recurrent VTE was defined as objectively verified progression of the thrombosis following start of treatment (18). CRNMB was defined by the recommended criteria by the International Society on Thrombosis and Haemostasis (21). Major bleeding was defined according to the Control of Anticoagulation Subcommittee as fatal bleeding, and/or symptomatic bleeding in a critical area or organ, and/or bleeding causing a fall of hemoglobin level of 2 g/dL or more, or leading to transfusion of two or more units of whole blood or red cells (22).

### 2.3 Statistics

The number of outcomes were counted for the control group and each group of patients taking the potentially interacting drugs. ORs and 95% confidence intervals (CI) were calculated, comparing the frequencies between the control group and interaction groups. An OR of 1 means no difference between groups of patients, an OR >1 means increased risk for the outcome, while and OR <1 means decreased risk for the outcome. If the 95% CI includes 1 it means that the odds ratio is not statistically significant. Descriptive statistics were used to summarize the baseline characteristics of the study population. We did simple counts of occurrences of categorical variables. Continuous data were expressed as means with standard deviation (SD) or median with range. Among the potentially interacting drugs, those used by five or more study patients were subject to further comparison of outcomes compared to the control group. Potentially interacting drugs used by 1-4 patients were only included in analysis of groups of drugs. IBM SPSS statistics software was used to do the statistical calculations.

## 3 Results

### 3.1 Drugs used by study participants

The median number of unique drugs taken per patient in addition to apixaban during the trial period was 13 (range 0-58).

A list of the selected individual drugs, the number of patients taking each drug and their expected interactions is detailed in **Supplementary Table 1**. Four patients were prescribed medications considered exclusion criteria during the study period (enzalutamide and dabrafenib) and were taken out of the study for safety reasons.

### 3.2 Baseline characteristics

The study population and subgroups are summarized in **Table 2**. Of the 298 enrolled patients, 26 (9%) patients experienced recurrent venous thrombosis, 42 (14%) had one or more CRNMB and 22 (7%) had a major bleeding during the 36-month study period. Upon reviewing the data, 2 patients had one or more CRNMB as well as a major bleeding, 4 patients had one or more CRNMB and a recurrent VTE. These 6 patients were included in both the CRNMB group and the major bleeding or recurrent VTE group, respectively.

**Table 2:** Endpoints and baseline characteristics for the different groups of interacting drugs.

	Overall study population	No suspected interacting drugs taken	Drugs expected to increase bleeding risk	Drugs expected to increase thrombosis risk	Drugs expected to increase both bleeding risk and thrombosis risk
Patients in group	298	74	16	8	200
Recurrent VTE	26	7	0	1	18
CRNMB *	42	10	2	1	29
Major bleeding	22	9	0	1	12
Age, years (mean ± SD)	66 ± 11	67 ± 12	73 ± 8	75 ± 5	65 ± 12
Platelet count (mean ± SD)	246 ± 110	257 ± 122	200 ± 53	260 ± 117	246 ± 108
BMI (mean ± SD)	24.5 ± 7.6	23.7 ± 8.5	25.6 ± 11.1	27.3 ± 4.4	24.6 ± 7.1
Female (%)	129 (43.4%)	35	3	6	85
Male (%)	169 (56.7%)	39	13	2	115
Diabetics (%)	44 (14.8%)	10	4	1	29
Previous VTE (%)	30 (10.1%)	11	4	1	14
Received antithrombotic prophylaxis before VT (%)	26 (8.7%)	9	1	0	16

VTE, venous thromboembolism; CRNMB, clinically relevant non-major bleeding; OR, odds ratio; CI, confidence interval; SD, standard deviation

* Of the 42 patients with CRNMB, 2 had major bleeding and 4 had recurrent VTE, and are thus also included in those groups.

Two hundred patients (67%) used drugs expected to reduce the anticoagulant effect of apixaban as well as drugs expected to increase the bleeding risk. Only 8 patients (3%) were exposed only to drugs suspected to reduce the anticoagulant effect of apixaban without any other known interfering drugs, while 16 patients (5%) received only one or more of the drugs with expected bleeding interactions. Finally, 74 patients (25%) did not use any of the suspected interactors.

### 3.3 Risk of bleeding and recurrent VTE for categories of interacting drugs

We did not find any association between categories of interacting drugs and the risk of recurrent VTE or any type of clinically relevant bleeding compared with patients who took no interacting drugs (**Table 3**).

**Table 3:** Odds ratios for recurrent VTE, CRNMB and major bleeding according to group of interacting drugs.

Outcomes	Control group (no known interactors taken) (N=74)	Expected increased bleeding risk only (N=16)	Expected increased thrombosis risk only (N=8)	Expected both increased bleeding risk and increased thrombosis risk (N=200)
Recurrent VTE (n)	7	0	1	18
No recurrent VTE (n)	67	16	7	182
OR recurrent VTE, 95% CI	1 (ref)	–	1.4 (0.15 – 13)	0.95 (0.38 – 2.4)
CRNMB (n)	10	2	1	29
No CRNMB	64	14	7	171
OR CRNMB, 95% CI	1 (ref)	0.91 (0.18 – 4.6)	0.91 (0.1 – 8.2)	1.1 (0.5 – 2.4)
Major bleeding (n)	9	0	1	12
No major bleeding (n)	65	16	7	188
OR major bleeding, 95% CI	1 (ref)	–	1.0 (0.11 – 9.4)	0.46 (0.19 – 1.1)

VTE, venous thromboembolism; CRNMB, clinically relevant non-major bleeding; OR, odds ratio; CI, confidence interval

### 3.4 Risk of recurrent VTE and bleeding for each drug

We found no association between use of any of the suspected drugs and recurrent VTE (**Table 4**). The highest point estimates of the OR for VTE were seen for aprepitant, gemcitabine, macrolide antibiotics, and NSAIDs, with broad confidence intervals. No OR could be calculated for bevacizumab as none of the 19 patients taking bevacizumab had a recurrent VTE.

**Table 4:** Odds ratio for recurrent venous thromboembolism.

Recurrent VTE				
Drug/group name	Number of patients	Recurrent VTE	No recurrent VTE	OR (95% CI)
No interactors taken	74	7	67	1 (ref)
Expected increased bleeding risk only				
Fluconazole	14	2	12	1.6 (0.3 – 8.6)
Macrolide antibiotics	10	2	8	2.4 (0.42 – 14)
Netupitant	34	3	31	0.93 (0.22 – 3.8)
Platelet inhibitors	38	4	34	1.1 (0.31 – 4.1)
Expected both increased bleeding risk and increased thrombosis risk				
Aprepitant	23	5	18	2.7 (0.75 – 9.4)
Bevacizumab	19	0	19	–
Glucocorticoids	188	16	172	0.89 (0.35 – 2.3)
NSAIDS	22	5	17	2.8 (0.79 – 10)
Expected increased thrombosis risk only				
Gemcitabine	19	4	15	2.6 (0.66 – 9.9)

VTE, venous thromboembolism; OR, odds ratio; CI, confidence interval; NSAIDS, non-steroidal anti-inflammatory drugs.

Regarding CRNMB (**Table 5**) and major bleedings (**Table 6**) we found no association with any drugs, except that the use of fluconazole perhaps was associated with CRNMB. Neither the use of NSAIDs nor antiplatelet therapy were associated with bleeding. No major bleeding was registered for the 10 patients taking macrolides nor for the 23 patients taking aprepitant, thus no OR could be calculated.

**Table 5:** Odds ratio for clinically relevant non-major bleeding.

CRNMB				
Drug/group name	Number of patients	1 or more CRNMB	No CRNMB	OR (95% CI)
No interactors taken	74	10	64	1 (def)
Expected increased bleeding risk only				
Fluconazole	14	5	9	3.6 (0.99 – 13)
Macrolide antibiotics	10	1	9	0.71 (0.08 – 6.2)
Netupitant	34	2	32	0.40 (0.08 – 1.9)
Platelet inhibitors	38	4	34	0.75 (0.22 – 2.6)
Expected both increased bleeding risk and increased thrombosis risk				
Aprepitant	23	2	21	0.61 (0.12 – 3.0)
Bevacizumab	19	4	15	1.7 (0.47 – 6.2)
Glucocorticoids	188	26	162	1.0 (0.47 – 2.3)
NSAIDS	22	3	19	1.0 (0.25 – 4.1)
Expected increased thrombosis risk only				
Gemcitabine	19	3	16	1.2 (0.3 – 4.87)

CRNMB, clinically relevant non-major bleeding; OR, odds ratio; CI, confidence interval

**Table 6:** Odd ratio for major bleeding.

Major bleeding				
Drug/group name	Number of patients	Major bleeding	No major bleeding	OR (95% CI)
Control group (no interactors taken)	74	9	65	1 (def)
Expected increased bleeding risk only				
Fluconazole	14	1	13	0.56 (0.06 – 4.8)
Macrolide antibiotics	10	0	10	–
Netupitant	34	2	32	0.45 (0.09 – 2.2)
Platelet inhibitors	38	1	37	0.2 (0.02 – 1.6)
Expected both increased bleeding risk and increased thrombosis risk				
Aprepitant	23	0	23	–
Bevacizumab	19	2	17	0.85 (0.17 – 4.3)
Glucocorticoids	188	23	165	1.0 (0.44 – 2.3)
NSAIDS	22	2	20	0.72 (0.14 – 3.6)
Expected increased thrombosis risk only				
Gemcitabine	19	1	18	0.4 (0.05 – 3.4)

OR, odds ratio; CI, confidence interval

## 4 Discussion

More than half of the cancer patients treated with apixaban for VTE in the current study used interacting drugs expected to increase the risk of thrombosis as well as bleeding. None of the potentially interacting drugs, or groups of drugs with similar interactions, were associated with recurrent VTE or clinically relevant bleeding. Although fluconazole perhaps increased the risk of CRNMB. Thus, a number of drugs expected to increase the risk of bleeding or thrombosis did not do so in our study. This included NSAIDs, platelet inhibitors, glucocorticoids, and bevacizumab. We found a high level of polypharmacy, with each patient taking a median of 13 different drugs at some time during the 36 month period.

Other studies of interaction with apixaban and other DOACs have focused on specific drugs or families of drugs, and most studies are done in the non-cancer population. In contrast, the current study investigated the clinical effects of interactions with all potentially interacting drugs used by cancer patients. A recent study by Wang et al. (17) looked at drug-drug interactions with DOACs or LMWH in cancer patients using LexiComp® data, grouping patients as either using interacting drugs or not. Like us, they found that many cancer patients used drugs interacting with anticoagulant treatment (42%), but there were no differences in recurrent VTE or bleeding events between patients with or without drug-drug interactions.

A 2013 study by Mueck et al. (23) found a significant increase in AUC and maximum drug concentration in plasma in co-administration of rivaroxaban and CYP3A4/P-gp inhibitors like fluconazole, erythromycin, and clarithromycin (23). In our study, fluconazole seemed to be associated with CRNMB, but not with major bleedings. Interestingly, we see that another study have found the same, apparently contradicting, result between CRNMB and major bleeding (24). We found no convincing effects on clinical outcomes for macrolides. As the number of patients taking macrolides was low, we did not perform analysis on the separate macrolide drugs. Within the group of macrolides, azithromycin is a weak interactor, leading to the possibility that it somewhat masked the interactions of stronger interactors in the macrolide group. A previous study assessed the bleeding risk of older adults taking a DOAC (apixaban, dabigatran or rivaroxaban) and either azithromycin and clarithromycin (25), and found an increased risk of hospital admission because of major bleeding in patients on clarithromycin compared with those taking azithromycin. In our study, none of the 10 patients taking macrolides experienced major bleeding.

There are many previous studies of interaction between DOACs and single drugs, some of those drugs were used by patients in our study. Verapamil and diltiazem have been investigated without finding any interaction with DOACs (15, 26, 27), while our study only had one patient taking each of these drugs, so we were not able to add any new information. The APPRAISE-2 trial found increased bleeding in patients with acute coronary syndromes taking apixaban with aspirin alone or aspirin and clopidogrel compared with placebo (28). The current study had 38 patients taking aspirin and two of those were also taking clopidogrel, but no association with CRNMB or major bleeding was found. A theoretical interaction between aprepitant and DOACs has been described (29), with the potential to cause both increased and decreased effect of anticoagulation, depending on the timing. Our results suggest that, if there is an effect, aprepitant may increase the risk of VTE and reduce the risk of bleeding since no patients taking aprepitant had major bleeding and only two patients had CRNMB, while five patients had recurrent VTE. Bevacizumab has previously been associated with a higher risk of VTE in cancer patients (30), while more recent studies indicate that it can protect against VTE (31). Our results are in line with the latter as none of the 19 patients taking bevacizumab experienced recurrent VTE.

Glucocorticoids are frequently used by cancer patients receiving chemotherapy. They are both associated with increased risk of VTE (32, 33) and with increased gastrointestinal bleeding when taken together with DOAC (34). In our study, 126 of the 298 participants used glucocorticoids. A convincing result was the lack of association with bleeding or recurrent VTE among these patients. The lack of bleeding risk may be related to prophylactic use of proton pump inhibitors.

There are several methodological challenges in investigating clinical effects of drug-drug interactions. One problem is that many drugs are used for short periods of time, e.g., antiemetic drugs, dexamethasone, or antifungal drugs. Thus, the increased risk of an adverse outcome is limited to a few days. We have defined users of interacting drugs as patients who used the interacting drug at any duration of time during the study, not factoring in the time for potential interactions to occur nor the timing in relation to endpoints. A second challenge are the patients who use drugs that can both increase and decrease the efficacy of apixaban and other drugs metabolized through CYP3A4 and P-pg. Thus, the sum of apparently contradicting effects is very difficult to estimate. On the other hand, the data from our study may indicate that polypharmacy could be beneficial, i.e., that the summation of the effects of drugs working opposite on the hemostatic system may reduce the risk of bleeding and recurrent VTE.

The main limitation of the current study is the low number of endpoint events, which resulted in broad CIs of a number of the risk estimates. Thus, there might be drugs increasing the risk of thrombosis or bleeding, where we found no association. Another possible limitation is that there may have been patients in what we have used as the control group, assumed to take no interacting drugs, but still doing so, who we did not consider in our analyses. In addition, we did not have information on intake of food that could possibly interact with apixaban. From the list of potentially interacting drugs, only gemcitabine, dabrafenib, enzalutamide, and tamoxifen were suspected to increase thrombosis risk without any increased bleeding risk. Of the 28 patients taking one or more of these drugs, 20 were also taking one or more drugs that could increase the risk of bleeding, leaving the group of “increased thrombosis risk” with only eight patients. This naturally caused lower confidence in any results from this group, due to the small size.

## 5 Conclusion

Apixaban interact with drugs inducing or inhibiting both CYP3A4 and P-gp. It is common among cancer patients to use drugs that potentially interact with apixaban. Despite this concern for drug-drug interaction, drugs with moderate or unknown interacting potential were not found to increase the risk for bleeding or recurrent VTE in cancer patients treated for VTE with apixaban. A possible exception was interaction with fluconazole, which perhaps increases the risk for CRNMB. The results of our study together with the results of previous studies, indicate that drug-drug interactions with drugs of moderate or unknown interacting potential is not a major problem in cancer patients treated with apixaban. We suggest that a possible explanation for this is polypharmacy where drugs with opposite interacting effects on bleeding or thrombosis neutralize each other.

## Data Availability

The raw data supporting the conclusions of this article will be made available by the authors, without undue reservation.
