# Supplementary material for: Effect of drug interactions with apixaban on clinical outcomes in cancer patients with venous thromboembolism
Source: Front Oncol. 2025 Jan 27;15:1520725. doi: 10.3389/fonc.2025.1520725 (PMC11807833; doi:10.3389/fonc.2025.1520725)
Supplement: Supplementary file 1 [file DataSheet1.pdf]

Supplemental table 1 - List of selected individual drugs or drug families, expected outcomes and the known or suspected mechanism of interaction. Constituents of groups are shown in cursive. It is overlap between patient groups since many patients used more than one drug.

Drug name	No. patients	Mechanism of interaction
**Expected increased bleeding**		
Amiodarone *	1	Weak CYP3A4 inhibitor (1)
Carvedilol *	4	Weak P-gp inhibitor (2)
Diltiazem *	1	Moderate CYP3A4 inhibitor (3)
Fluconazole	14	Moderate CYP3A4 inhibitor (4)
Macrolides	10	
Azithromycin	*4*	P-gp inhibitor, unknown degree (5)
Clarithromycin	*2*	Weak CYP3A4 and P-gp inhibitor (6, 7)
Erythromycin	*4*	Known bleeding risk (6, 7)
Netupitant	34	Moderate CYP3A4 inhibitor (8)
Platelet inhibitors	38	Platelet inhibition, bleeding risk (9, 10)
ASA	*38*	
Clopidogrel	*2*	
Verapamil *	1	Weak CYP3A4 (11) and P-gp inhibitor (12)
**Expected reduced anticoagulant effect/increased thrombosis risk**		
Dabrafenib *	2	Strong CYP3A4 inducer (13)
Enzalutamide *	2	Strong CYP3A4 inducer (14)
Gemcitabine	19	Known thrombosis risk (15, 16)
Tamoxifen *	4	Known thrombosis risk (17)
**Expected both increased bleeding and reduced anticoagulant effect/increased thrombosis risk**		
Aprepitant	23	Moderate CYP3A4 inhibition during treatment (18)
		Transient mild CYP3A4 induction after treatment (18)
Bevacizumab	19	Known bleeding risk (19)
		Known thrombosis risk (20)
Glucocorticoids	188	Mucosal ulcers, platelet inhibition (COX pathway), increased risk of GI bleeding when taken with DOACs (21)

Dexamethasone	*137*	Known thrombosis risk (22, 23)<br>Known bleeding risk (16)<br>Moderate CYP3A4 inducer (24)
Prednisolone	*61*	
Methylprednisolone	*28*	
Hydrocortisone	*6*	
Cortisone	*5*	
Betamethasone	*1*	
NSAIDs	22	GI bleeding (25), platelet aggregation inhibition (26)<br>Unknown thrombosis mechanism (27)
Ibuprofen	*9*	
Diclofenac	*9*	
Naproxen	*2*	
Ketorolac	*1*	
Ketoprofen	*1*	
Indomethacin	*1*	

* Due to low number of patients taking the drug, analysis was not performed on the individual drug, but patients taking the drug were placed in the appropriate subgroup and thus excluded from the control group.

References in supplemental table 1

1. Ha HR, Candinas R, Stieger B, Meyer UA, Follath F. Interaction between amiodarone and lidocaine. Journal of cardiovascular pharmacology. 1996;28(4):533-9.

2. Wessler JD, Grip LT, Mendell J, Giugliano RP. The P-glycoprotein transport system and cardiovascular drugs. Journal of the American College of Cardiology. 2013;61(25):2495-502.

3. Sutton D, Butler AM, Nadin L, Murray M. Role of CYP3A4 in human hepatic diltiazem N-demethylation: inhibition of CYP3A4 activity by oxidized diltiazem metabolites. Journal of Pharmacology and Experimental Therapeutics. 1997;282(1):294-300.

4. Katz H. Drug interactions of the newer oral antifungal agents. The British journal of dermatology. 1999;141:26-32.

5. Gupta S, Banfield C, Kantesaria B, Marino M, Clement R, Affrime M, Batra V. Pharmacokinetic and safety profile of desloratadine and fexofenadine when coadministered with azithromycin: a randomized, placebo-controlled, parallel-group study. Clinical therapeutics. 2001;23(3):451-66.

6. Li A, Li MK, Crowther M, Vazquez SR. Drug-drug interactions with direct oral anticoagulants associated with adverse events in the real world: a systematic review. Thrombosis research. 2020;194:240-5.

7. Westphal JF. Macrolide–induced clinically relevant drug interactions with cytochrome P-450A (CYP) 3A4: an update focused on clarithromycin, azithromycin and dirithromycin. British journal of clinical pharmacology. 2000;50(4):285.

8. Lanzarotti C, Rossi G. Effect of netupitant, a highly selective NK1 receptor antagonist, on the pharmacokinetics of midazolam, erythromycin, and dexamethasone. Supportive Care in Cancer. 2013;21(10):2783-91.

9. Coukell AJ, Markham A. Clopidogrel. Drugs. 1997;54(5):745-50; discussion 51.

10. Evans G, Packham MA, Nishizawa EE, Mustard JF, Murphy EA. The effect of acetylsalicylic acid on platelet function. The Journal of experimental medicine. 1968;128(5):877-94.

11. Backman JT, Olkkola KT, Aranko K, Himberg J-J, Neuvonen PJ. Dose of midazolam should be reduced during diltiazem and verapamil treatments. British journal of clinical pharmacology. 1994;37(3):221-5.

12. Huang M, Liu G. The study of innate drug resistance of human hepatocellular carcinoma Bel7402 cell line. Cancer letters. 1998;135(1):97-105.

13. Puszkiel A, Noé G, Bellesoeur A, Kramkimel N, Paludetto M-N, Thomas-Schoemann A, et al. Clinical pharmacokinetics and pharmacodynamics of dabrafenib. Clinical Pharmacokinetics. 2019;58(4):451-67.

14. Gibbons JA, de Vries M, Krauwinkel W, Ohtsu Y, Noukens J, van der Walt J-S, et al. Pharmacokinetic drug interaction studies with enzalutamide. Clinical pharmacokinetics. 2015;54(10):1057-69.

15. Qi WX, Lin F, Sun YJ, Tang LN, Shen Z, Yao Y. Risk of venous and arterial thromboembolic events in cancer patients treated with gemcitabine: a systematic review and meta-analysis. British Journal of Clinical Pharmacology. 2013;76(3):338-47.

16. Barni S, Labianca R, Agnelli G, Bonizzoni E, Verso M, Mandalà M, et al. Chemotherapy-associated thromboembolic risk in cancer outpatients and effect of nadroparin thromboprophylaxis: results of a retrospective analysis of the PROTECHT study. Journal of Translational Medicine. 2011;9(1):1-7.

17. Meier CR, Jick H. Tamoxifen and risk of idiopathic venous thromboembolism. British journal of clinical pharmacology. 1998;45(6):608-12.

18. Dando TM, Perry CM. Aprepitant. Drugs. 2004;64(7):777-94.

19. Hang XF, Xu WS, Wang JX, Wang L, Xin HG, Zhang RQ, Ni W. Risk of high-grade bleeding in patients with cancer treated with bevacizumab: a meta-analysis of randomized controlled trials. European journal of clinical pharmacology. 2011;67(6):613-23.

20. Nalluri SR, Chu D, Keresztes R, Zhu X, Wu S. Risk of venous thromboembolism with the angiogenesis inhibitor bevacizumab in cancer patients: a meta-analysis. Jama. 2008;300(19):2277-85.

21. Holt A, Blanche P, Zareini B, Rasmussen PV, Strange JE, Rajan D, et al. Gastrointestinal bleeding risk following concomitant treatment with oral glucocorticoids in patients on non-vitamin K oral anticoagulants. Heart. 2022;108(8):626-32.

22. Johannesdottir SA, Horváth-Puhó E, Dekkers OM, Cannegieter SC, Jørgensen JOL, Ehrenstein V, et al. Use of glucocorticoids and risk of venous thromboembolism: a nationwide population-based case-control study. JAMA internal medicine. 2013;173(9):743-52.

23. Orsi FA, Lijfering WM, Geersing GJ, Rosendaal FR, Dekkers OM, le Cessie S, Cannegieter SC. Glucocorticoid use and risk of first and recurrent venous thromboembolism: self-controlled case-series and cohort study. British journal of haematology. 2021;193(6):1194-202.

24. McCune JS, Hawke RL, LeCluyse EL, Gillenwater HH, Hamilton G, Ritchie J, Lindley C. In vivo and in vitro induction of human cytochrome P4503A4 by dexamethasone. Clinical Pharmacology & Therapeutics. 2000;68(4):356-66.

25. Olsen A-MS, Gislason GH, McGettigan P, Fosbøl E, Sørensen R, Hansen ML, et al. Association of NSAID use with risk of bleeding and cardiovascular events in patients receiving antithrombotic therapy after myocardial infarction. Jama. 2015;313(8):805-14.

26. Rao G, Johnson GG, Reddy KR, White JG. Ibuprofen protects platelet cyclooxygenase from irreversible inhibition by aspirin. Arteriosclerosis: An Official Journal of the American Heart Association, Inc. 1983;3(4):383-8.

27. Varga Z, rafay ali Sabzwari S, Vargova V. Cardiovascular risk of nonsteroidal anti-inflammatory drugs: an under-recognized public health issue. Cureus. 2017;9(4).
